# Identification of Mycoviruses in *Cytospora chrysosperma*: Potential Biocontrol Agents for Walnut Canker

**DOI:** 10.3390/v17020180

**Published:** 2025-01-26

**Authors:** Yingjie Mi, Shaohua Chen, Kexin Liu, Zhanjiang Tie, Junchao Ren, Mingli Zhang, Zheng Liu, Sifeng Zhao, Hui Xi, Xuekun Zhang

**Affiliations:** 1 Key Laboratory of Oasis Agricultural Pest Management and Plant Protection Resources Utilization, College of Agriculture, Shihezi University, Shihezi 832003, China; 15630077211@163.com (Y.M.); 19108506803@163.com (K.L.); t2424395514@163.com (Z.T.); 15603583303@163.com (J.R.); zhangmingli4128@163.com (M.Z.); lzh8200@126.com (Z.L.); zhsf_agr@shzu.edu.cn (S.Z.); 2 Xinjiang Jiangjun Weinong Biotechnology Development Co., Ltd., Shihezi 832003, China; csh4581918@163.com

**Keywords:** walnut canker, *C. chrysosperma*, mycoviruses, biocontrol

## Abstract

Walnut canker is a common disease in the Xinjiang Uygur autonomous region of China, which is caused by *Cytospora chrysosperma*. To date, there is no effective control measure for this disease. Infection with mycoviruses has been widely proven to reduce the virulence of plant pathogenic fungi, with some mycoviruses even serving as potential biological control agents for plant diseases. In this study, mycoviruses associated with 31 strains of *C. chrysosperma* from Xinjiang Uygur autonomous region were identified by metatranscriptomic sequencing. Seven new mycoviruses were identified by BLAST and RT-PCR analysis, which were Botrytis cinerea partitivirus 5 (BcPV5), Gammapartitivirus sp-XJ1 (GVsp-XJ1), Botoulivirus sp-XJ2 (BVsp-XJ2), Luoyang Fusar tick virus 2 (LfTV2), Leptosphaeria biglobosa narnavirus 17 (LbNV17), Sclerotinia sclerotiorum narnavirus 6 (SsNV6), and Cytospora ribis mitovirus (CrMV3). Among these, BcPV5, GVsp-XJ1, BVsp-XJ2, CrMV3, and LfTV2 were found to co-infect *C. chrysosperma* strain WS-11 and significantly reduce both the colony growth rate and virulence of the host. After co-culturing the virus-free WS-FV strain with WS-11, the colony growth rate and virulence of the derivative strain were also decreased. These results provide potential biocontrol resources for the control of walnut canker.

## 1. Introduction

As an economic tree with excellent fruit and wood, walnut provides edible nuts, high-quality wood, and medicinal products [1,2], and is widely planted in southern regions of Xinjiang Uygur autonomous region, China [3]. However, walnut canker has seriously harmed the growth of walnut trees. The incidence of walnut canker has become increasingly severe due to the lack of effective prevention and control measures [4]. Walnut canker is mainly caused by *C. chrysosperma*, which primarily damages the cortex of tree branches and trunks, causing phloem decay and impacting the growth and yields [5]. Walnut canker is currently controlled mainly by removing diseased branches and applying fungicides [6]. However, no effective control method has been established for walnut canker in practical production. In recent years, many studies have found a large number of mycoviruses in fungi, some of which were used as biological control agents [7]. Identifying the mycoviruses in *C. chrysosperma* and screening for strains carrying mycoviruses with biological control potential is crucial for developing effective, environmentally friendly management strategies to control walnut canker.

Mycoviruses were widely distributed in all major fungal groups [8]. Most of the mycoviruses had double-stranded RNA (dsRNA) or positive-sense single-stranded RNA (+ssRNA) genomes, but there were also negative-sense single-stranded RNA (-ssRNA) or single-stranded DNA (ssDNA) [9]. The mycoviruses with dsRNA genomes were classified into seven families: *Chrysoviridae*, *Partitiviridae*, *Reoviridae*, *Megabirnaviridae*, *Totiviridae*, *Quadriviridae*, and *Endornaviridae* [10]. Some pathogens carrying mycoviruses have been used as biological control agents due to their hypovirulence [11]. For example, the Cryphonectria hypovirus 1 (CHV1) has been successfully used to control chestnut wilt disease caused by *Cryphonectria parasitica* in Europe [12]. Rosellinianecatrix megabirna virus 1 (RnMBV1) and Mycoreovirus 3 (MyRV3) reduced the virulence of heterologous strains through exogenous transformation [13]. However, there are few reports on the study of mycoviruses present in pathogens inciting walnut canker. Thus, understanding mycovirus transmission and mechanisms in other fungal pathogens offers valuable insights for mycovirus-based control of walnut canker.

Some mycoviruses have been shown to significantly alter the virulence of their fungal hosts. Sclerotinia sclerotiorum hypovirus 1 (SsHV1) significantly reduces the virulence of the host by interfering with host gene expression and metabolic pathways, thereby altering the production of secondary metabolites such as oxalic acid, which is crucial for the virulence of *Sclerotinia sclerotiorum* [14]. Furthermore, the *ORF*1 of Sclerotinia sclerotiorum negative-stranded RNA virus 1 (SsNSRV-1) regulates the transcription, translation, and modification of host genes, promoting viral proliferation while reducing host virulence. Additionally, Stemphylium lycopersici alternavirus 1 (SIAV1) induces hypovirulence by modulating the host’s RNA interference (RNAi) pathway, thereby suppressing fungal growth and virulence [15]. Mycoviruses could also reprogram fungal metabolism, leading to the production of antifungal compounds that inhibit competing pathogens [16]. Sclerotinia sclerotiorum hypovirulence-associated DNA virus 1 (SsHADV-1) could transform the pathogenic fungus *S. sclerotiorum* into endophytic fungi that could engage in mutualistic symbiosis with rape, promoting the growth of rape and enhancing plant disease resistance [17].

While some mycoviruses demonstrate promising biocontrol potential, it is also important to consider the mechanisms of mycovirus transmission and the factors influencing their spread within and between fungal populations. The transmission of mycoviruses typically occurs vertically from parent to offspring through hyphal fusion or spore formation, or horizontally among vegetatively compatible hosts [18]. Some mycoviruses can inhibit fungal non-self recognition, facilitating horizontal transmission between incompatible hosts and even across different fungal species [19]. There is a synergistic relationship between tobacco mosaic virus (TMV) and mycovirus CHV1, which can increase cross-kingdom infections between plants and fungi. TMV can enhance the transmission of CHV1 from infected fungal cells to plant tissues, while CHV1 can facilitate the infection and replication of TMV within the fungal host [20]. Sclerotinia sclerotiorum mycoreovirus 4 (SsMYRV4) has been shown to reduce vegetative incompatibility in *S. sclerotiorum* by inhibiting the expression of heterotrimeric guanine nucleotide-binding proteins (G proteins) and nutrient-affinity related genes, thereby preventing programmed cell death (PCD) [21].

In this study, the mycoviruses in 31 strains of *C. chrysosperma* isolated from Xinjiang Uygur autonomous region were analyzed by sub-transcriptome sequencing. The mycoviruses in each strain were analyzed using BLAST and RT-PCR. The impact of the mycoviruses on host growth and virulence was determined indoors, and attenuated strains were selected from them. The results of this study could provide new insights and biological control resources for the prevention and control of walnut canker.

## 2. Materials and Methods

### 2.1. Strains of C. chrysosperma

A total of 31 strains of *C. chrysosperma* were provided by Professor Zheng Liu from our research group. Among them, 12 strains were isolated from Xinhe County, Xinjiang Uygur autonomous region (designated as XH), and 19 strains were isolated from Wensu County, Xinjiang Uygur autonomous region (designated as WS). All strains were cultured on potato dextrose agar (PDA) in a 25 °C incubator and stored at −80 °C in 25% glycerol. The colony morphology was recorded on the seventh day. Meanwhile, the mycelia were collected after 3–5 d of culture and immediately placed in liquid nitrogen to extract RNA.

### 2.2. Metatranscriptomic Sequencing and Bioinformatics Analysis

*C. chrysosperma* strains were classified into groups A (18 strains) and B (13 strains) based on colony morphology and growth rate. Approximately 12,500 ng of RNA from each group was used for metatranscriptomic sequencing based on the Illumina MiSeq 2500 platform (Beijing Novogene Bioinformatics Technology Co., Ltd., Beijing, China). The Epicenter Ribo-ZeroTM Kit (Epicentre, Madison, WI, USA) was used to deplete rRNA. A fragment buffer was used to induce random disruption of RNA, followed by synthesis of the first cDNA strand using a random hexamer. The cDNA library was purified with AMPure XP beads (Merck, Darmstadt, Germany) and amplified by PCR. De novo assembly of clean reads was performed using IDBA_UD with various k-mer values to generate contigs. These sequences were filtered against rRNA, tRNA, and SILVA databases, with subsequent BLAST searches against the NCBI non-redundant protein sequence database applying an e-value threshold of ≤1 × 10^−3^. The lowest common ancestor (LCA) algorithm was used for classification, and contigs linked to the virus branch were further analyzed with BLASTx (BLAST+ 2.13.0) to identify viral types. Homologous virus information was retrieved from NCBI using BLASTx. Contigs with over 90% similarity to known viruses were considered as different strains of the same virus.

### 2.3. Putative Mycovirus Confirmation

The cDNA was synthesized from the RNA using the EasyScript One-Step gDNA Removal and cDNA Synthesis SuperMix reverse transcription kit (TransGen, Beijing, China) following the manufacturer’s protocol. The putative viral cDNA was identified by Reverse Transcription Polymerase Chain Reaction (RT-PCR) using specific primers (Table S1) designed using Premier 5.0. The RT-PCR reaction mixture consisted of 10 µL of 2× Taq Master Mix, 1 µL each of the upstream and downstream primers, 1 µL of cDNA, and 7 µL of ddH_2_O.

### 2.4. Growth Rate and Pathogenicity Test

A mycelial agar plug (5 mm in diameter) was selected from the colony edge of the strains cultured for 5 d and placed on a 9-cm-diameter PDA plate. Then, the culture was incubated in the dark at 25 °C for 5 d, after which the mycelial growth rate was measured and the colony morphology was observed. The virulence of each strain was determined by virulence test. Briefly, branches were inoculated with agar plugs of actively growing mycelia, then placed in a styrofoam chamber that was subsequently covered with a plastic membrane to maintain a constant humid atmosphere (90% relative humidity) at 25 °C in a constant-temperature incubator. Noncolonized PDA plates were also inoculated and incubated in parallel with the controls. The lesions that developed from the inoculated branches were measured and photographed at the fifteenth day.

### 2.5. Horizontal Transmission of Hypovirulence

The WS-11, carrying five mycoviruses (CrMV3, BcPV5, GVsp-XJ1, BVsp-XJ2, and LfTV2), and virus-free *C. chrysosperma* WS-20 (WS-FV) strains were chosen for co-cultivation. The activated donor strain WS-11 was inoculated on a PDA plate about 1.5 cm from the edge of the culture dish and cultured for 2 d. Subsequently, the recipient strain WS-FV was placed 1.5 cm away from the donor strain, and WS-11 was used as a control. Each treatment was repeated 4 times, and the experiment was repeated 2 times. The WS-11 strain was co-cultured with WS-FV on PDA culture dishes (9 cm in diameter) at 25 °C for 10 d. Following co-cultivation, a mycelial agar plug from the periphery of the WS-FV colony was transferred to a new PDA plate, generating three derivative strains (WS-FV-V) from a location 4 cm away from the side nearest to the recipient strain. Then, the derivative strains were cultured on a cellophane-layered PDA plate for 4 d, after which the RNA was extracted and the presence of the mycoviruses were confirmed by RT-PCR.

## 3. Results

### 3.1. Identification of Walnut Canker Pathogens

**Figure 1:**
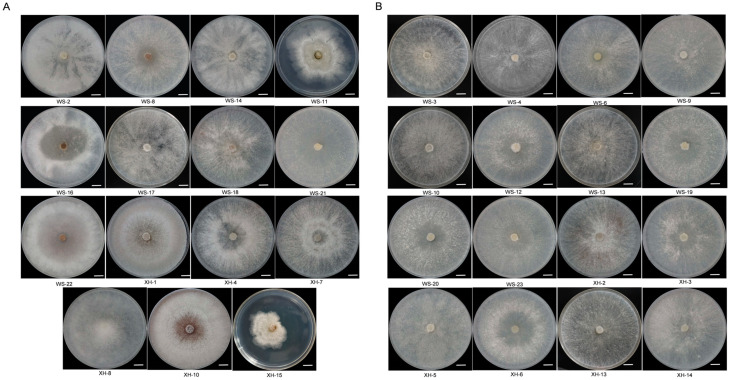
Colony morphology of *C. chrysosperma* (25 °C, 7 d). (**A**) *C. chrysosperma* with abnormal colony morphology (bars = 1 cm). (**B**) *C. chrysosperma* with normal colony morphology (bars = 1 cm).

**Figure 2:**
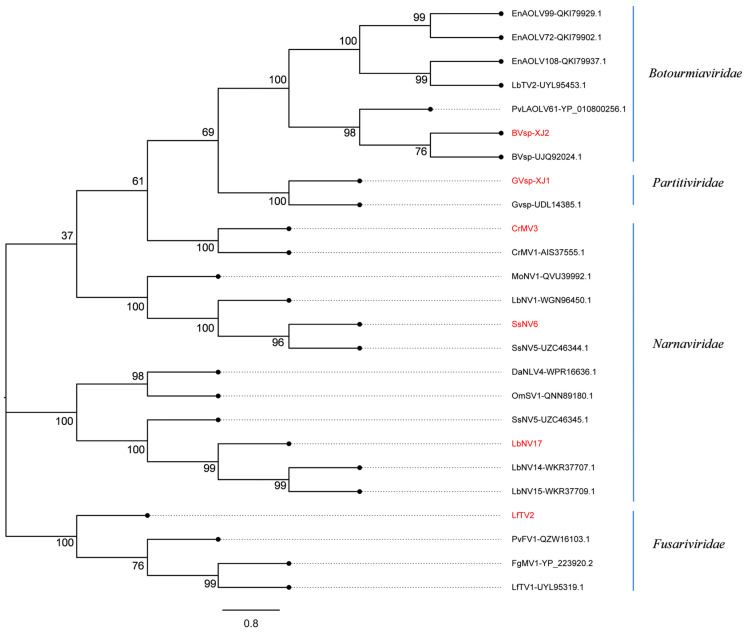
The phylogenetic tree of mycoviruses in *C. chrysosperma* constructed based on RdRp amino acid sequences. The phylogenetic tree was generated using MEGA 7.0 software with the Maximum Likelihood (ML) algorithm and 1000 bootstrap replicates. The mycoviruses identified in *C. chrysosperma* are highlighted in red.

To identify mycoviruses in *C. chrysosperma* strains from Xinjiang Uygur autonomous region, we categorized the 31 preserved laboratory strains into two groups to streamline the sequencing process: (1) low colony growth, abnormal colony morphology, sparse hyphae, and irregular colony edges (Figure 1A); (2) fast colony growth, dense mycelial growth, and regular colony edges (Figure 1B).

### 3.2. Metatranscriptomic Identification of Mycoviruses Infecting the Tested Strains

More than 21 Gb of raw reads were generated by Metatranscriptomic sequencing, from which a total of 17,122 high-quality clean reads were derived after removing joints and low-quality reads. A total of 13 contigs representing partial genomic regions of 7 mycoviruses were identified by BLASTx analysis against the NCBI database. Specifically, one contig’s amino acid sequence showed similarity to Botrytis cinerea partitivirus 3 and was designated as Botrytis cinerea partitivirus 5 (BcPV5). Two contigs exhibited similarity to *Gammapartitivirus* sp. and were named *Gammapartitivirus* sp.-XJ1. Four contigs were similar to *Botoulivirus* sp. and were designated as *Botoulivirus* sp.-XJ2. Two contigs exhibited similarity to Luoyang Fusar tick virus 1 and were named Luoyang Fusar tick virus 1. One contig’s sequence was similar to Leptosphaeria biglobosa narnavirus 14 and was named Leptosphaeria biglobosa narnavirus 17. One contig’s sequence was similar to Sclerotinia sclerotiorum narnavirus 5 and was named Sclerotinia sclerotiorum narnavirus 6. Lastly, two contigs showed similarity to *Cytosporaribis mitovirus* 1 and were named *Cytosporam ribis* mitovirus 3 (Table 1).

### 3.3. Phylogenetic Analysis

The results of phylogenetic analysis revealed that CrMV3, SsNV6, and LbNV17 were closely affiliated with the family *Narnaviridae* based on the RNA-dependent RNA polymerase (RdRp) sequence, forming distinct branches and being recognized as novel members within this family. Furthermore, BVsp-XJ2 exhibited a close relationship with BVsp (UJQ92024.1) from the family *Botourmiaviridae*. GVsp-XJ1 was closely related to GVsp (UDL14385.1) from the family *Partitiviridae*. LfTV2 was categorized into a separate branch within the family *Fusariviridae* and characterized as a new mycovirus (Figure 2).

**Figure 3:**
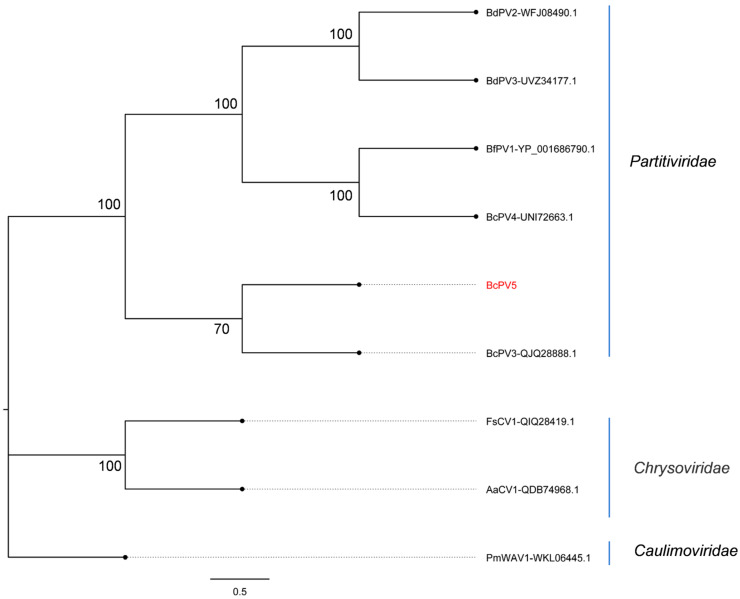
The phylogenetic tree of the putative mycovirus BcPV5 conducted based on the CP amino acid sequence. The phylogenetic tree was generated using MEGA 7.0 software with the Maximum Likelihood (ML) algorithm and 1000 bootstrap replicates. The mycovirus identified within *C. chrysosperma* is highlighted in red.

On the other hand, contig113 (BcPV5) corresponded to the capsid protein (CP) amino acid sequences of the mycovirus by BLASTx analysis against the NCBI database. The phylogenetic analysis results of the CP amino acid sequences from BcPV5 and other members of the family *Partitiviridae* demonstrated that BcPV5 shared the highest similarity with BcPV3, with a sequence identity of 44.72%. Consequently, BcPV5 is proposed as a new member of the family *Partitiviridae* (Figure 3).

### 3.4. Detection of Mycoviruses and Their Impact on the Growth of C. chrysosperma

**Figure 4:**
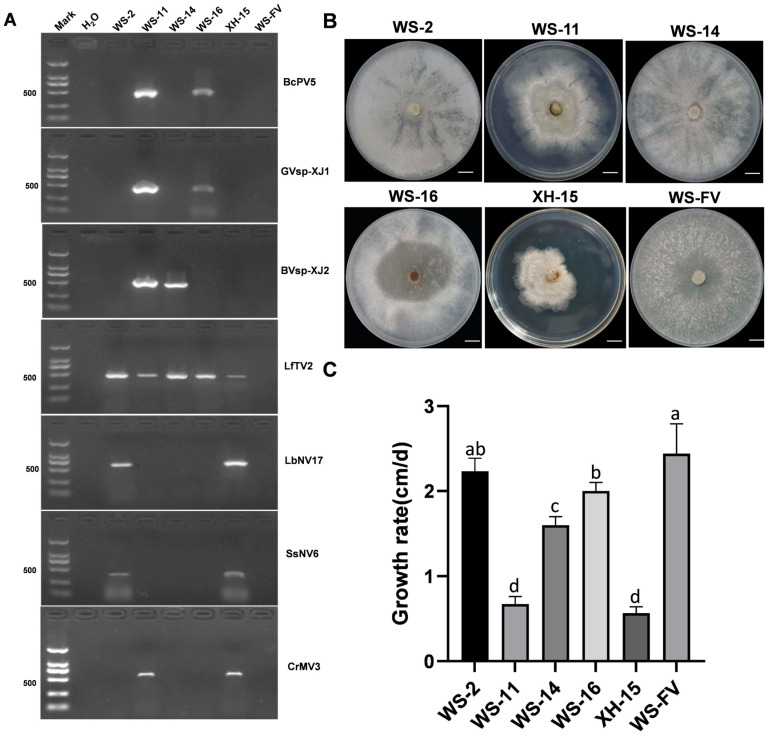
Detection of seven mycoviruses and their impact on the growth of *C. chrysosperma.* (**A**) Detection of the seven mycoviruses in the *C. chrysosperma* strains, with ddH_2_O serving as the negative control. (**B**) The colony morphology of *C. chrysosperma* on PDA plates incubated in the dark at 25 °C for 5 d (bars = 1 cm). (**C**) The growth rate of *C. chrysosperma* strains incubated in the dark at 25 °C for 5 d. Data are presented as mean ± standard deviation (SD) of four replicates. Different letters represent significant differences in *p* < 0.01 using one-way analysis of variance (ANOVA).

To elucidate the distribution of these seven mycoviruses within the isolated strains of *C. chrysosperma*, we extracted the total RNA and employed specific primers to identify the putative mycovirus by RT-PCR (Table S1). The results showed that seven mycoviruses were detected in 29 out of 31 strains (Figure S1). Compared to the control strain WS-FV, strains WS-2, WS-11, WS-14, WS-16, and XH-15 exhibited abnormal colony morphology (Figure 4B), which harbored three, five, two, three, and four mycoviruses, respectively (Figure 4A). However, strains WS-9 (LfTV2), WS-10 (SsNV6), XH-11 (SsNV6), WS-13 (LfTV2 and SsNV6), XH-3, XH-6, and XH-14 (LbNV17 and SsNV6) had no obvious morphological differences with WS-FV. Strain WS-2, carrying mycoviruses LfTV2, LbNV17, and SSNV6, displayed altered colony morphology but a similar growth rate to WS-FV. In contrast, strains WS-14 (BVsp-XJ2 and LfTV2) and WS-16 (BcPV5, GVsp-XJ1, and LfTV2) showed significant reductions in growth rate and increased aerial hyphae, respectively. Most notably, strains WS-11 and XH-15, both infected with BVsp-XJ2 and CrMV3, experienced significant changes in colony morphology and a substantial decrease in growth rate (Figure 4B,C). These results suggested that mycoviruses GVsp-XJ1, LfTV2, LbNV17, and SsNV6 had no significant impact on *C. chrysosperma*, while BVsp-XJ2 and CrMV3 were pivotal in affecting both colony morphology and growth rate.

### 3.5. Virulence Determination of Strains Carrying Mycoviruses

**Figure 5:**
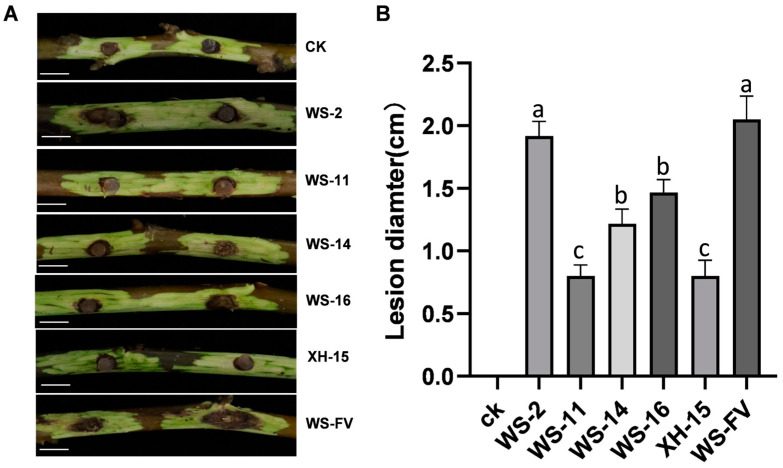
Virulence determination of the strains carrying mycoviruses. (**A**) Virulence of the strains carrying mycoviruses on detached walnut branches 15 d after inoculation (bars = 1 cm). (**B**). The lesion length of the strains carrying mycovirus on detached walnut branches 15 d after inoculation. CK was inoculated with noncolonized PDA plugs. The data are presented as means ± SD of four replicates. Different letters represent significant differences in *p* < 0.01 using ANOVA.

To ascertain the impact of mycoviruses on the virulence of *C. chrysosperma*, the virulence was determined. The results revealed that WS-11, WS-14, WS-16, and XH-15 not only displayed inhibited host growth, but also diminished host virulence. The lesion diameters of these strains were significantly smaller than those of WS-FV (Figure 5A). Specifically, WS-11 and XH-15 exhibited the smallest lesion diameter on detached branches, with a 60.98% reduction compared to WS-FV, followed by WS-14 and WS-16 with decreases of 40.49% and 28.39%, respectively. In contrast, the lesion diameter of WS-2 did not significantly differ from that of WS-FV (Figure 5B).

### 3.6. Horizontal Transmission of Mycoviruses

**Figure 6:**
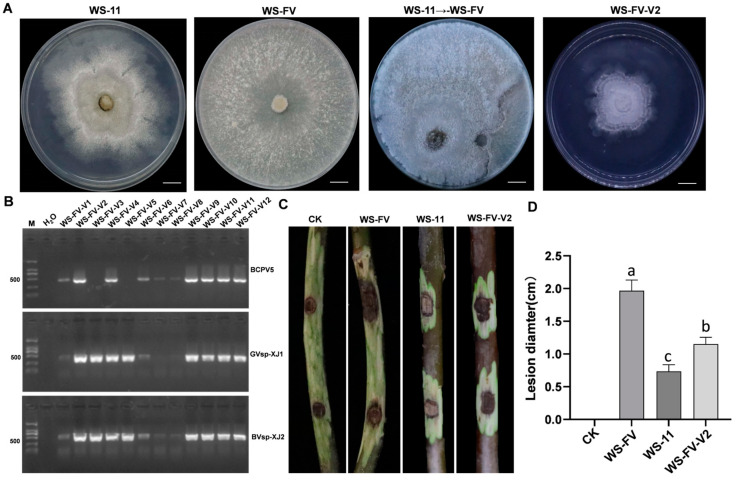
Horizontal transmission of mycoviruses and its impact on the virulence of *C. chrysosperma*. (**A**) The colony morphology of *C. chrysosperma* strains cultured for 5 d (bars = 1 cm). (**B**) Detection of mycoviruses in derivative WS-FV-V strains. (**C**) The lesions formed by WS-11, WS-FV, and WS-FV-V2 strains on detached walnut branches 15 d after inoculation. (**D**) The lesion diameter of detached walnut branches caused by WS-11, WS-FV, and WS-FV-V2 strains 15 d after inoculation. CK was inoculated with noncolonized PDA plugs. The data are presented as means ± SD of four replicates. Different letters represent significant differences in *p* < 0.01 using ANOVA.

To assess the transmission ability of mycoviruses carried by hypovirulence strain WS-11 to other *C. chrysosperma* strains, WS-11 was co-cultured with the recipient strain WS-FV. The derivative strains near WS-FV were selected to detect mycovirus transmission by RT-PCR. The results revealed that BcPV5, GVsp-XJ1, and BVsp-XJ2 could be transmitted from WS-11 to WS-FV, with transmission efficiencies of 83.33%, 88.33%, and 100%, respectively. In contrast, CrMV3 and LfTV2 were not detected in the derivative strains (Figure 6A,B). The derivative strains obtained were divided into three types: one carrying only GVsp-XJ1 and BVsp-XJ2, another carrying only BcPV5 and BVsp-XJ2, and a third carrying all three mycoviruses (GVsp-XJ1, BVsp-XJ2, and BcPV5). Among these, the derivative strain WS-FV-V2, which harbored all three mycoviruses, was selected for virulence assessment. The virulence of WS-FV-V2 on detached branches was significantly diminished, resulting in fewer lesions compared to the WS-FV (Figure 6C,D). Notably, the presence of GVsp-XJ1, BVsp-XJ2, and BcPV5 significantly affected the colony morphology of the derivative strains and reduced the virulence.

## 4. Discussion

Metatranscriptome sequencing technology has significantly broadened our comprehension of mycovirus diversity and facilitated the discovery of novel mycoviruses within plant pathogenic fungi. In this study, a total of seven novel mycoviruses were identified in 31 *C. chrysosperma* strains by metatranscriptomic sequencing. Notably, the hypovirulent WS-11 strain carrying mycoviruses demonstrated substantial biocontrol potential. Furthermore, the virulence of derivative strain WS-FV-V2 was significantly reduced upon co-cultivation with virus-free *C. chrysosperma* strains. These findings not only elucidate the species of mycoviruses among the pathogens causing walnut canker, but also offer valuable biological control resources for managing walnut canker.

Mycoviruses are ubiquitous in fungi and often cause asymptomatic infections, but can also alter fungal characteristics such as mycelial growth, spore production, virulence, pigmentation, and secondary metabolism [22]. These changes can reduce the virulence of plant pathogenic fungi, suggesting their potential use as biological control agents [23]. For example, SlAV1 from *Stemphylium lycopersici* modifies colony pigmentation and virulence [15], and dsRNA viruses can disrupt essential processes like cell wall synthesis and pigment biosynthesis in *C. chrysosperma* [24]. Mycoviruses may also significantly impact fungal growth rate, spore production, and virulence, possibly by interfering with host gene expression and metabolic pathways [25]. In this study, we found that BVsp-XJ2 and CrMV3 altered *C. chrysosperma*’s colony morphology and decreased its virulence.

Fungi can harbor a variety of viral species. Co-infections with multiple mycoviruses are common in fungi [26]. *C. chrysosperma* can be infected by various mycoviruses, including double-stranded and single-stranded RNA mycoviruses [24]. The YkV1 mycovirus could enhance YnV1 mycovirus accumulation in *R. necatrix* strain W97, and co-infections in strain W1032 were associated with growth defects, indicating a symbiotic relationship between YkV1 and its viral partner [27]. Similarly, *C. parasitica* carried multiple mycoviruses from at least six families, causing diverse symptoms or asymptomatic infections [28]. This study found that WS-11 and XH-15 carried five and four mycoviruses, respectively, and the mycoviruses also affected host growth and reduced virulence. However, the mechanisms behind these synergistic effects require further study.

The development of mycoviruses as biocontrol agents for managing agricultural diseases has good potential, especially in sustainable agriculture [29]. Mycoviruses can help reduce the severity of plant diseases by infecting and weakening the virulence of pathogenic fungi [30]. For example, hypovirulence has been observed in several species such as *S. sclerotiorum* and *B. cinerea*, where specific mycoviruses diminish host virulence [31,32]. This phenomenon provides a foundation for developing novel biological control strategies. Moreover, mycoviruses can also control disease by inducing low virulence in pathogenic fungi [33]. For instance, the application of VIGS (virus-induced gene silencing) vectors targeting endogenous genes has successfully transformed fungi into low virulence strains, significantly decreasing the incidence of wheat *Fusarium* head blight [34]. On the other hand, vegetative incompatibility could impede the dissemination and application of mycoviruses [35]. However, recent studies have shown that exogenous addition of proline could overcome transmission barriers between vegetatively incompatible fungal strains, thereby enhancing crop yields [36].

Mycoviruses are transmitted vertically from mycelia to spores and horizontally through hyphal anastomosis, with transmission efficiency being crucial for evaluating their potential as biological control agents [37]. In this study, the strain WS-11, carrying five mycoviruses, and its derivative strains all exhibited obvious hypovirulence. Among the transmitted mycoviruses, three mycoviruses with high transmission efficiency were detected in the derivative strains, among which BVsp-XJ2 exhibited the highest transmission rate, reaching 100%. This finding provides a scientific basis for the future development of mycoviruses as biocontrol agents against walnut canker caused by *C. chrysosperma*. However, further research is needed to elucidate the reasons behind the transmission failure of LfTV2 and CrMV3 to the WS-FV strain, particularly focusing on the interaction mechanisms between these mycoviruses and their host. Since this study has identified several mycoviruses with biocontrol potential, substantial research is still required to effectively implement these mycoviruses in field applications. Further studies should focus on elucidating the mechanisms of mycovirus transmission, optimizing their application methods, and evaluating their long-term effects on crop health and yield. These efforts will be crucial for translating mycovirus-based biocontrol strategies from laboratory research to practical agricultural use.

## Figures and Tables

**Table 1:** Best BLASTx matches of the contigs obtained.

Number	Contig Number	Contig Length	Name of Putative Viruses	Best Match	aa Identity	Genome Type	Family/Genus
1	Acontig113	1456 bp	Botrytis cinerea partitivirus 5 BcPV5	Botrytis cinerea partitivirus3 QJQ28888.1	44.72%	dsRNA	*Partitiviridae*
2	Acontig729	924 bp	Gammapartitivirus sp.-XJ1 GVsp-XJ1	*Gammapartitivirus* sp. UDL14413.1	78.18%	dsRNA	*Partitiviridae*
3	Acontig372	2116 bp	Botoulivirus sp-XJ2 BVsp-XJ2	*Botoulivirus* sp. UJQ92024.1	52.89%	+ssRNA	*Botourmiaviridae*
4	Acontig412	6806 bp	Luoyang Fusar tick virus 2 LfTV2	Luoyang Fusar tick virus 1 UYL95319.1	50.82%	+ssRNA	*Fusariviridae*
5	Acontig397	2486 bp	Leptosphaeria biglobosa narnavirus 17 LbNV17	Leptosphaeria biglobosa narnavirus 14 WKR37707.1	68.16%	+ssRNA	*Narnaviridae*
6	Acontig519	2566 bp	Sclerotinia sclerotiorum narnavirus 6 SsNV6	Sclerotinia sclerotiorum narnavirus 5 UZC46344.1	70.60%	+ssRNA	*Narnaviridae*
7	Acontig526	3116 bp	Cytosporam ribis mitovirus 3 CrMV3	Cytosporaribis mitovirus 1 AIS37555.1	64.67%	+ssRNA	*Narnaviridae*

## Data Availability

The data presented in this study are available in the article.

## Supplementary Materials

The following supporting information can be downloaded at: https://www.mdpi.com/article/10.3390/v17020180/s1, Table S1: The primers used in this paper; Figure S1: Mycovirus distribution among 31 strains of *Cytospora chrysosperma*.
