# Supplementary material for: Identification of Mycoviruses in Cytospora chrysosperma: Potential Biocontrol Agents for Walnut Canker
Source: Viruses. 2025 Jan 26;17(2):180. doi: 10.3390/v17020180 (PMC11860865; doi:10.3390/v17020180)
Supplement: Supplementary file 1 [file viruses-17-00180-s001.zip › viruses-3411524-supplementary.pdf]

Supplementary Materials

Table S1. The primers used in this paper.

Name	Sequence (5'→3')	Purpose	Product size
A1BV353F	CGACGAGTTGATGTAGTTCT	Botrytis cinerea partitivirus 5	354bp
A1BV353R	TCCTTCTTCAACTGCATCTC		
A2GV317F	GGTGAAATCCTTCATGGTCT	Gammapartitivirus sp.-XJ1	317bp
A2GV317R	TGACACAACGCTAAGTAACA		
A3BV557R	GGAACGGATGATGCGAGGAT	Botoulivirus sp-XJ2	387bp
A3BV387F	TCTCCCCGAGTATGCACTCA		
A4LV305F	CCAGTGAATGTGTCTAACCA	Luoyang Fusar tick virus 2	305bp
A4LV305R	GCAAAGACTAAGAACCCAGA		
A5LV557F	TGCTTTGCCCAACATTCGTG	Leptosphaeria biglobosa narnavirus 17	557bp
A5LV557R	GGAACGGATGATGCGAGGAT		
B1SV328F	CGTACGGGATAAGGTCGGTG	Sclerotinia sclerotiorum narnavirus 6	328bp
B1SV328R	GCCAGCATTCATTGTAGCGG		
B2CV529F	GGCCCGAGATCTTCAATAAT	Cytosporam ribis mitovirus 3	529bp
B2CV529R	TTCGTTTTCTCTGAAGAGGG		

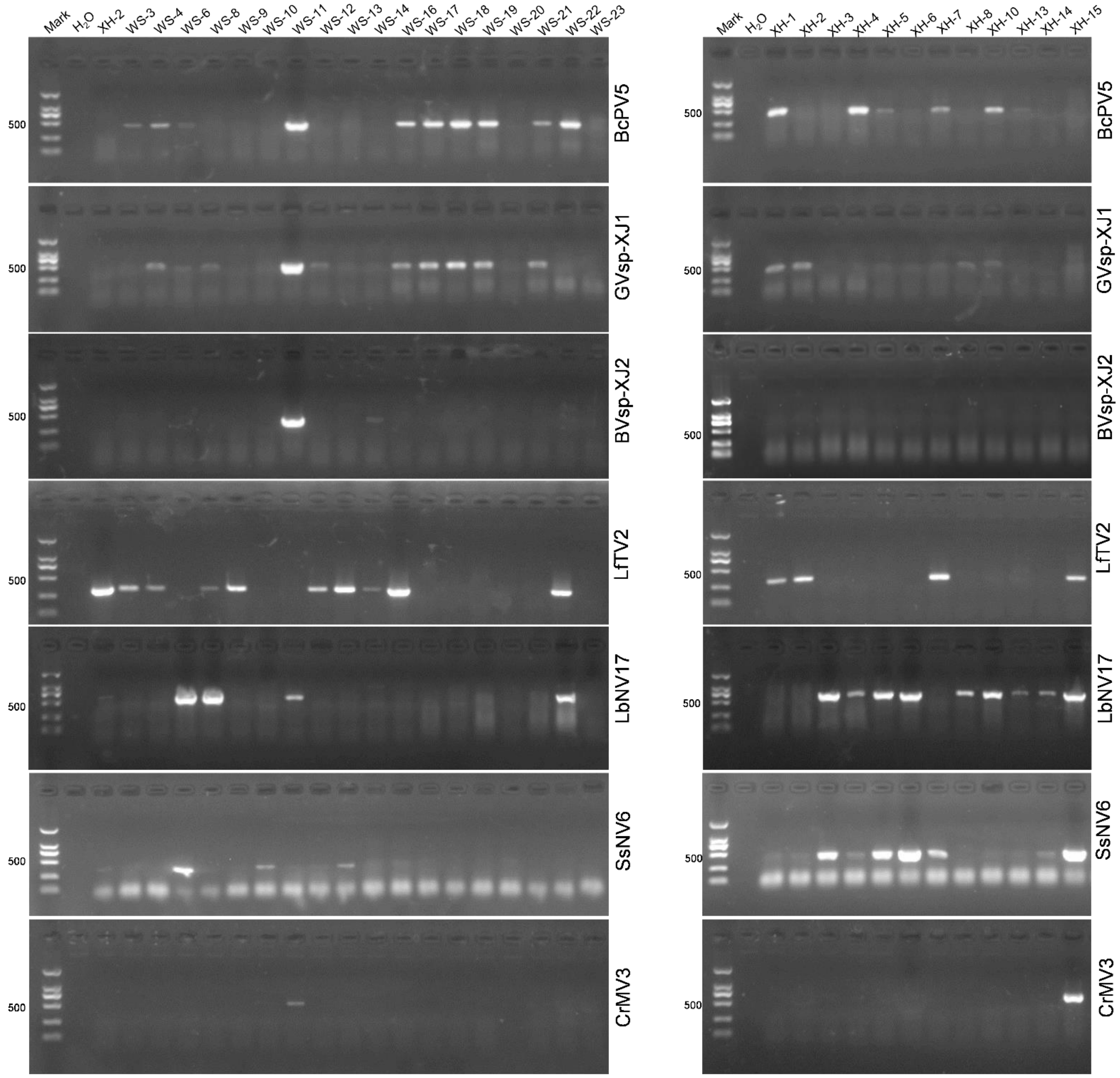

Figure S1. Mycovirus distribution among 31 *C. chrysosperma* strains.
